# The possible prevention of cancer

**DOI:** 10.1186/1476-069X-10-S1-S13

**Published:** 2011-04-05

**Authors:** John Cairns

**Affiliations:** 1 Clinical Trial Service Unit, University of Oxford, Oxford OX3 7LF, UK and 105 Alleyn Park, London SE21 8AA, UK

## Abstract

The prevention of the infectious diseases was accomplished long before there was any understanding of the molecular biology of bacteria and viruses. As for cancer, the sharp drop in frequency of the once-commonest lethal cancer, stomach cancer, was achieved without any contribution from biological research, and the current drop in lung cancer is the end-result of the observation by epidemiologists that most lung cancer is caused by smoking. So the basis for both these triumphs was essentially empirical and owed nothing to biological research. This paper discusses how molecular biology can now offer the possibility of large-scale protection against cancer.

## Article

Research into cancer has in the past been largely managed by doctors and the emphasis has tended to be on finding new treatments rather than on prevention. But it is quite clear that life expectancy in the industrialised world had almost doubled by the time the first antibiotics were discovered [1], and this must therefore have been achieved by prevention of the major infectious diseases rather than by devising better forms of treatment. As for cancer, the two main successes against the common cancers, namely the declines in lung and stomach cancer, were not achieved by treatment but by deliberate or fortuitous prevention, respectively.

Cancers arise largely or wholly as the result of mutation, each kind of cancer being commonly associated with particular combinations of mutations in genes known to be involved in the regulation of cellular behaviour [2]. Furthermore, the same genes are involved in experimental carcinogenesis in mice. Recently it has become possible to scan the entire genome of cancer cells, and this has shown that the genomes of patients’ cancers usually differ by many hundreds of changes from the genomes of the patients’ normal cells [3]. Incidentally, the high mutation rate (and therefore versatility) of cancer cells may explain why it has been so hard to find a drug that kills all the cells in a patient’s cancer.

One of the major projects in cancer research is to look for inherited mutations in human populations that raise the risk of cancer, particularly the risk of breast cancer because this is the common cancer that tends to run in families. Unfortunately, apart from two mutant genes long known to be associated with a high risk of breast cancer, the search has shown that changes in a large number of genes influence overall risk and so this knowledge may have little practical application.

The early days of molecular genetics showed, however, that you learn about the pathway to an end-result by studying mutations that block that pathway and that you may learn little or nothing from the mutations that accelerate it. For example, early in the 20^th^ century it was Garrod’s and Cunéot’s studies of the “inborn errors of metabolism” that disclosed the pathway which breaks down phenylalanine and tyrosine in humans [4] and makes the pigments in mouse skin [5], and that was the start of the science of molecular genetics. If we want to learn about the pathway to cancer and perhaps learn how to prevent people from developing cancer we should be looking for mutations that *lower* the risk of cancer rather than just those that raise risk.

The project could not easily be carried out in humans. Although, there are hints that a few people may have a lower risk of cancer than the average, the evidence is unavoidably weak since only about 50% of the human population develop cancer. But at least it is clear that mice do vary in their susceptibility to experimental carcinogenesis [6] and that it is possible by selective breeding to create groups of mice with either raised or lowered susceptibility [7]; (the latter exercise came to nothing because, at the time, the main object was to create mice with raised susceptibility so that they could be used as a tester strain for carcinogens). The present project should therefore start with a study in mice and then, if successful, be extended to humans. (A possible shortcut would be to look in the existing data bases for alleles that are significantly more common in old people who have never developed cancer [8].

Returning now to the origins of humankind, it is clear that our world is far less demanding and dangerous than it used to be, and the best of all possible genomes in ancient times is unlikely to be the best for today. No doubt the same is true for the pampered laboratory mouse. Therefore a search for resistance genes, first in mice and then in humans, seems a reasonable project. In recent years it has become even more plausible, because a new ingredient has emerged for understanding the workings of natural selection. Many years ago, bacteria were observed to react to stress by increasing their mutation rate [9]. This “SOS response” as it was called [10] was found to be dependent on the activity of certain genes involved in DNA repair that are not involved in the normal growth and low mutation rate of bacteria. Later these findings were extended to yeast and mammalian cells, and a whole host of genes are now known whose products cope with all kinds of damage (in particular, the damage caused by heat-shock), including some protection against damaged and defective proteins. From the point of view of cancer research, the most important feature of the response is that once induced, for example by DNA damage, it can switch on the high mutation rate for several cell generations [11]. And this, incidentally, offers us a new way of interpreting the strange stages observed in experimental carcinogenesis – namely, that “*initiation*” by mutagenic carcinogens may be simply the induction of what in mammalian cells is usually called the heat-shock response, and that the subsequent prolonged process of “*promotion*” is simply the disruption of tissue organization which then allows the incursion of natural selection and the preferential survival of the fittest mutants.

Speculations about the mechanisms underlying biological processes usually prove to be vast oversimplifications, because they underestimate the complexity of biology. Happily, the present argument is not dependent on correctness of interpretation. For it is already clear that inactivation of the heat-shock response (by knocking out both copies of the gene, HSF1, that switches on the response) greatly lowers the susceptibility of mice to induced and spontaneous cancers, without at the same time much affecting their viability and longevity [12]. So we do know that polymorphisms can exist that lower the risk of cancer, and it is imaginable that one day some form of genetic manipulation may be used as a general preventive measure against most cancers. (A possible shortcut to such a lengthy program could be to use the sequencing data accumulated in the search for alleles that raise susceptibility and look for polymorphisms in human HSF1 that are rare in old people who have had cancer).

## Conclusion

There is, I think, an important message from this very brief history. The prevention of the infectious diseases was accomplished long before there was any understanding of the molecular biology of bacteria and viruses. As for cancer, the sharp drop in frequency of the once-commonest lethal cancer, stomach cancer, was achieved inadvertently and without any contribution from biological research, and the current drop in lung cancer is the much-delayed end-result of the observation by epidemiologists that most lung cancer is caused by smoking. So the basis for both these triumphs was essentially empirical and owed nothing to biological research. Perhaps now the time has come for modern molecular biology to make the breakthrough.

## Competing interests

The author declares that he has no competing financial or non-financial interests.
